# Prevalence of *Anaplasma phagocytophilum *in ticks collected from migratory birds in Danube Delta, Romania

**DOI:** 10.1186/1756-3305-7-S1-P16

**Published:** 2014-04-01

**Authors:** ID Mărcuţan, AD Sándor, AD Mihalca, CM Gherman, Z Kalmár, G D'Amico, MO Dumitrache, V Cozma

**Affiliations:** 1 Department of Parasitology and Parasitic Diseases, University of Agricultural Sciences and Veterinary Medicine, Cluj-Napoca, Romania

## 

Wild birds are hosts for several species of ticks, contributing to the maintenance of their local populations in delimited geographic areas. migratory birds play important roles as distributors of ticks within and between distant territories, including continents. Ticks collected from birds are responsible for hosting a significant number of human pathogens. The extensive wetland complex of the Danube Delta provides an internationally important stopover site for millions of birds, belonging to 300 different species, travelling annually. The aim of this study was to detect *Anaplasma phagocytophilum *in feeding ticks collected from migratory birds along four migratory seasons. Ticks were collected from the birds with forceps and preserved in 96% ethanol for later examination using a separate vial for each bird. A total of 1436 birds in 56 species (15 families) of Passeriformes and 3 non-Passeriformes bird species were captured. A total of 400 ticks were collected and identified as larvae (n = 191; 47.75%), nymphs (n = 201; 50.25%) or adult females (n = 8; 2%). No adult males were found. The ticks belonged to four species (*Ixodes ricinus, I. arboricola, I. redikorzevi *and *Haemaphysalis punctata)*.

*Ixodes ricinus *was the most common tick (369/400 ticks, 92.25% of the total collected), with a total of 181 larvae, 180 nymphs and 8 females. For detection of *Anaplasma phagocytophilum *all 400 ticks were examined by PCR, targeting the *msp2 *gene. *Anaplasma phagocytophilum *specific DNA was detected in 2 larvae and 2 nymphs of, *I. ricinus*, from 3 birds prevalence 1.08-%. The infection was absent from the other species of collected ticks

The bird species that carried ticks infected with *A. phagocytophilum *were *Turdus merula, Erithacus rubecula *and *Fringilla coelebs*. Several other studies have demonstrated the presence of *A. phagocytophilum *in ticks collected from birds. Comparing our data with these results, we confirm the low prevalence of *A. phagocytophilum*, showingthat migratory birds, despite being without reservoir competencemight be important hosts for the dispersal of infected ticks.

## Funding

This research was supported by grant CNCSIS IDEI PCCE 7/2010 and IDEI PCE 236/2011.

